# Regression models for the prediction of the influence of magnesium ions on primary endothelial cell (HUVEC) proliferation and migration

**DOI:** 10.1016/j.csbj.2025.06.023

**Published:** 2025-06-08

**Authors:** Heike Helmholz, Redon Resuli, Marius Tacke, Jalil Nourisa, Sven Tomforde, Roland Aydin, Regine Willumeit-Römer, Berit Zeller-Plumhoff

**Affiliations:** a Institute of Metallic Biomaterials, Helmholz-Zentrum Hereon, Geesthacht, Germany; b Institute of Material Systems Modeling, Helmholz-Zentrum Hereon, Geesthacht, Germany; c Department of Computer Science, Faculty of Engineering, Kiel University, Kiel, Germany; d Institute for Continuum and Material Mechanics, Hamburg University of Technology, Hamburg, Germany; e Kiel, Nano, Surface, and Interface Science - KiNSIS, Kiel University, Germany; f Data-driven Analysis and Design of Materials, Faculty of Mechanical Engineering and Marine Technologies, University of Rostock, Germany; g Department Life, Light & Matter, Interdisciplinary Faculty, University of Rostock, Germany

**Keywords:** Regression models, HUVEC, Cell proliferation, Large Language Model

## Abstract

Angiogenesis is one of the first stages in fracture healing and bone repair. Therefore, numerous studies evaluating the effect of Mg as a promising degradable, metallic biomaterial on the proliferation and function of endothelial cells have been performed. However, these studies lack methodological homogeneity and therefore differ in fundamental conclusions. Here, Mg-concentration-, donor- and cell age- dependent relations to primary human umbilical cord vein endothelial cells (HUVEC) proliferation and migration were investigated systematically. The generated data were utilized to develop regression models in order to assess and predict the cell response on Mg exposition in a concentration range of 2–20 mM Mg in cell culture medium extract. A concentration of > 2 mM already induced a detrimental effect in the sensitive primary HUVECs. Molecular data quantifying angiogenesis markers supported this finding. An increased migration capacity has been observed at a concentration of 10 mM Mg. We compared linear regression, random forests, support vector machines, neural networks and large language models for the prediction of HUVEC proliferation for a number of scenarios. Using these machine learning methods, we were able to predict the proliferation of HUVECs for missing Mg concentrations and for missing passages with mean absolute errors below 10 % and as low as 8.5 %, respectively. Due to strong differences between the cell behaviour of different donors, information for missing donors can be predicted with mean absolute errors of 15.7 % only. Support vector machines with linear kernel performed best on the tested data, but large language models also showed promising results.

## Graphical Abstract

**Figure:**
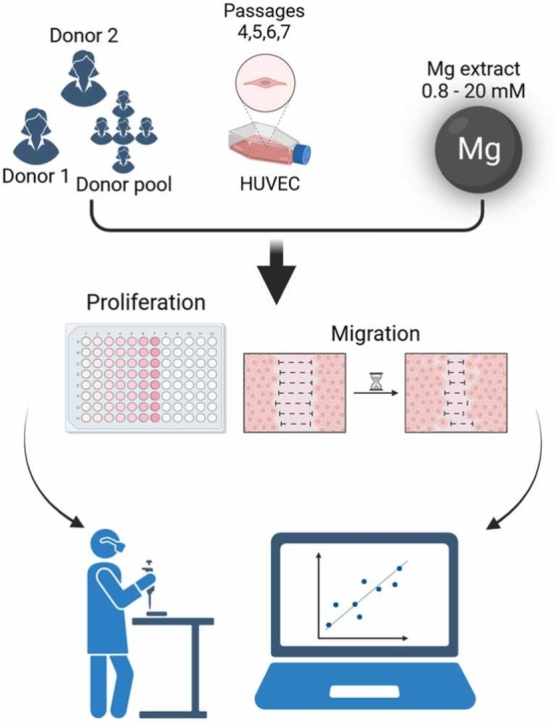

## 1 Introduction

Angiogenesis, the development of blood vessels from existing ones, is an essential process during fracture repair and tissue regeneration to ensure the supply of cells with oxygen and nutrients. Furthermore, the recruitment of tissue repair cells (stem cells, immune cells) requires intact blood vessels. This process is one of the first steps in the orchestrated course of fracture repair mechanism [1]. A tissue trauma can induce a hypoxic condition leading to the production of the transcription factor hypoxia inducible factor 1 (HIF-1) which consequently triggers the release of vascular endothelial growth factor A (VEGF-A), an essential signal for sensitive endothelial cells to change the morphology and develop filopodia to migrate and transform into the tips of new vessels. Furthermore, proliferation of endothelial cells is favoured to build the elongation of branches. However, angiogenesis is also involved in pathological processes such as diabetic retinopathy or vascularisation of cancer metastasis. The induction of angiogenesis is a major component in particular for the progression of neoplastic diseases. Therefore suitable *in vitro* models and tools to study angiogenesis processes are required to investigate potential regulators of angiogenetic responses either as agonists or antagonists [2].

There are several options to describe and investigate the potential effects of degradable implants in general and Mg in particular, especially on the viability and function of endothelial cells. In order to achieve a comprehensive overview, it is essential to monitor not only the viability but also the functionality in the form of migration assays. An evaluation of underlying processes can be performed at a molecular level utilizing gene and protein expression patterns. Consequently, we targeted the question of which types of data are feasible to develop predictive models to replace or support complex cell culture approaches. There is a range of studies describing the impact of Mg on the behaviour of endothelial cells. Banai et al. found that Mg up to 2.4 mM increases the proliferation and migration of bovine adrenal cortex capillary endothelial cells [3]. Maier et al. and Bernardini et al. confirmed these results with human endothelial cells up to a concentration of 10 mM Mg [4], [5]. Xu et al. found HUVEC promoting effects at Mg concentrations below 8 mM [6]. Liu et al. defined 5 mM Mg to be optimal for cultivating primary HUVECS [7]. A systematic review by Salandova et al. summarizes not only the applicable analytical assays but elucidates the effect of five different inorganic biomaterial additives. In this context Mg was not as efficient in promoting angiogenesis as strontium [8]. However, Mg-containing biomaterial are of special interest as temporary cardiovascular stents requiring a smooth compatibility with inner cell layer of vessels [9]. The assessment of the impact of Mg in all these studies depends on material-related parameters such as Mg source, cell-related parameters as cell type, age and furthermore analytical factors such as applied test methods, their reliability and sensitivity. Very often no specific information on these parameters can be found in the literature. This heterogeneity makes comparison and correlation of conclusions challenging.

Therefore, it is desired to build regression models that enable the prediction of cell culture test outcomes for different parameters. Specifically, if predictive mathematical models of drug/stimuli effects on different cells such as HUVEC can be developed, testing of new treatment strategies can be moved from *in vitro* experiments to *in silico* experiments [10]. This is desired to increase the amount of testing that can be performed while minimizing the required resources. Previously, mathematical models have been used to predict HUVEC proliferation dependent on certain drug concentrations [11]. A more established field for mathematical modelling is the modelling of tumour cell proliferation [12]. In addition to mathematical modelling, which is mechanism-based, machine learning models may be used for regression purposes. In a recent work, these different approaches were implemented to assess their ability to predict tumour cell proliferation over time, highlighting a comparable predictability [13]. Mehrian et al. [14] recently used machine learning based models to predict the population doubling time of human mesenchymal stromal cells based on donor characteristics, such as age, gender and passage number. They showed that random forests displayed great potential to assist in cell culture test. Shen et al. [15] used gradient boosting decision trees to predict mouse osteoblast-like cell proliferation on titanium dioxide nanotubes and found that cell seeding density and sterilization methods had the potential to not only affect but invert the proliferation behaviour.

The aim of this study is to evaluate the use of different regression models for the prediction of HUVEC proliferation response to Mg concentrations, when certain amounts of information are unknown. For this purpose, we contribute to the state of the art by testing how we can use traditional regression models, specifically linear regression, support vector regression and random forests, to predict the HUVEC proliferation for a wide range of Mg concentrations, not available passages or missing donor information. If successful, we may be able to draw comparisons between published experiments with different testing parameters without the necessity for further experimental work. In addition to the traditional regression models, we are implementing neural networks and further compare to the use of large language models (GPT-4o and o4-mini), in order to assess the benefit that the use of such a broadly trained model may bring for a scientific domain-specific problem. In addition to providing the experimental data on the HUVEC proliferation itself, we also provide data on HUVEC migration.

## 2 Materials and methods

### 2.1 Material extract preparation

Magnesium extracts were prepared utilizing Mg (99,95 % purity) casted material (Helmholtz Zentrum hereon) according to iso 10993–5 and as previously described in [16]. The surfaces of the Mg cubs were cleaned by grinding and submersing in organic solvents (n-hexan, acetone and 100 % ethanol) each 20 min in ultrasound bath (Branson 1210, Branson, Connecticut, USA). The extracts were prepared in a ratio of 0.2 g solid material to 1 mL basic Endothelial cell growth medium 2 (ECGM 2) without supplements by incubating under physiological conditions (37°C, 5 % CO_2_ and humidified atmosphere) for 72 h. The Mg concentration was determined by AAS (Agilent 240 AA; Agilent Technologies, Waldbronn, Germany) in a concentration range of 0.5–1.0 mg/L (single element reference solution; Carl Roth, Karlsruhe, Germany). An air/acetylene flame type with a pre-read delay of 3 s and a triplicate read of 3 s read time each was applied for the element specific detection at a wavelength of 285.2 nm [17]. The Mg concentration was required to standardize the applied assay concentrations and to control the quality of the extracts.

### 2.2 Cell culture

Primary endothelial cells were prepared from umbilical cord vein as described in [6]. Ethical approval for the isolation of HUVECs was obtained from the Ethik-Kommission der Ärztekammer, Hamburg (PV5991). Umbilical cord samples were provided by Bethesda Hospital Bergedorf (Hamburg, Germany) after caesarean sections of consenting donors. Two different donors were compared with a commercial donor pool (Promocell, Heidelberg, D).

The cells were continuously cultured in Endothelial cell growth medium 2 (ECGM2, Promocell Heidelberg, Germany) with supplements (Promocell, Heidelberg, Germany) under physiological conditions 37 °C, 5 % CO_2_ and humidified atmosphere. The passage representing the age of the culture was counted with the initial primary cryo-conserved cells as 1. The first subculturing after reactivation was used for expansion before the assays could start with passage 3.

### 2.3 Proliferation assay

To determine the growth of HUVECs under the impact of Mg degradation products, the proliferation rate was determined via a viability assay cell counting kit 8 (CCK8, Boster Biological Technology Co, Ltd, Pleasanton, USA). Cells were seeded in a density of 5000 cells/well in a flat bottom 96-well plate and incubated in complete ECGM 2 for 24 h. After this adaptation phase the Mg extracts were applied diluted to the required concentration (2 – 20 mM) with complete ECGM2. The cell viability was measured after 3 days incubation under physiological conditions by adding a volume of 10 µL CCK8 test solution followed by an incubation of 2 h. The colour development was measured by a multiplate reader (Victor 3, Perkin Elmer, USA) at a wavelength of 450 nm. The experiments were performed with 8 technical replicates and a blank reference as the extract containing medium was used for data assessment.

### 2.4 Migration assay

The scratch or migration assay was chosen as functional assay. Two concentrations of 5 and 10 mM Mg were selected, and each experiment was performed in a 24-well plate with 4 technical replicates. A density of 50,000 cells were seeded per well and incubated for three days in complete ECGM2 until an approximated confluency of 90 % was achieved. After this period the cells were kept in ECGM2 medium without supplements and further treated with 500 µL of 10 µg/mL mitomycin C solution for 2 h to inhibit the proliferation and target the migration of cell [18]. The cells were washed three times with PBS and after the first washing a scratch was set at the well surface manually using a 100 µL pipette tip. The remaining two washing steps were applied to remove the cell debris from the scratch. The first set of images were taken immediately after setting the scratch as T0 and furthermore after 3, 6 and 9 h (T3, T6 and T9). For image acquisition and analysis, a Nikon Eclipse TI (Nikon instruments, Melville, USA) was used together with MONO NIS Elements AR software (version 5.20.000) to define the same starting points for each time point for an automated acquisition. Image quantification was performed with ImageJ (version 1.52 t.22.08.22) either manually or with the MRI_wound_healing_tool (MRI_Wound_Healing.ijm).

To quantitatively analyse the migration data, the average closed wound area in % per well for T3-T0, T6-T0, and T9-T0 was computed by first calculating the area change for each position measured and then averaging per well. However, during microscopy of migration tests for donor 2 passage 4 and the donor pool passage 7, there was a discrepancy between measured positions. Therefore, the results of all areas in the four respective wells were averaged prior to determination of the area change. Donor 2 passage 7 and donor pool passage 5 were missing due to technical issues with the microscope. Thus, only passages 4 and 6 were available for all donors. Following averaging, linear functions were fitted to determine the area change over time per passage number and donor and Mg concentration, which can be interpreted as a measure of migration capacity. Due to the low number of samples, the migration data was not used for regression analysis.

### 2.5 Gene expression

The Quantitative Reverse Transcriptase Polymerase chain (qRT-PCR) reaction was applied to determine the expression of genes in HUVEC exposed to Mg degradation products at 5 and 10 mM Mg concentrations in parallel to the proliferation assay. HUVECs were seeded in 6- well plates 1.5 × 10^5^ cells/well and cultured in ECGM2 for 24 h before adding the Mg extracts. The cells were exposed to Mg for further 3 days. Cells growing in complete ECGM2 serves as control. The RNA extraction of the cells was performed with the Quick-RNA Microprep (Zymo, CA, USA) according to the manual and the RNA concentration and purity were determined utilizing nanodrop2000. The isolated RNA was utilized to synthesize complementary cDNA (ReverdAid, Thermo fisher, Germany). The qRT-PCR was performed using PerfeCTa SYBR® Green FastMix (QuantaBio, MA, USA). Each sample was tested in triplicates. The gene expression was quantified with a CFX Opus96 thermocycler (BioRad, Munich, Germany) in triplicates. The values of all controls independent of the passage number were combined as control in order to perform a 2 delta CT (ΔΔ CT) method (software CFX Maestro and CFX Manager (version 3.0)). The reference genes for the RT PCR were beta Actin (*ACTB*) and beta 2 macroglobulin (*B2M)*. Genes of interest related to angiogenesis were Angiogenin (ANG), VEGF-A, VEGF-B, Vascular endothelial growth factor receptor 1 (*FLT1*) and Vascular endothelial growth factor receptor 2 (*KDR*). The applied primer sequences were documented in Table 1.

**Table 1:** List of reference and tested genes with the corresponding primer sequences and length.

**Name of the indicator (gen)**	**Forward primer sequence 5’-3’** **Reverse primer sequence 5’-3’**	**Length**
Angiogenin *(ANG)*	CATCATGAGGAGACGGGG TCCAAGTGGACAGGTAAGCC	264
Vascular endothelial growth factor A (*VEGF-A*)	CCCTGATGAGATCGAGTACATCTT ACGCTCCAGGACTTATACCG	300
Vascular endothelial growth factor B (*VEGF-B*)	GCTCTTCTGCCATCCCTTGT TAGTGAGGGGAGGAAGAGCC	180
Vascular endothelial growth factor receptor 1 (*FLT1*)	GGGCTGAAACCATGTGCAAG GCCAAAGATGCACTCCTCCT	165
Vascular endothelial growth factor receptor 2 (*KDR*)	GGCATGGGGTCTGTTCTGAA TTGGCCAGGAGACACGTAAC	155
Beta−2-microglobulin (*B2M*)	CTTTCTGGCCTGGAGGCTATC AGACCAGTCCTTGCTGAAAGA	201
Beta actin (*ACTB*)	CTTCCTGGGCATGGAGTC TGATCTTCATTGTGCTGGGT	188

### 2.6 Statistics

Using the statsmodels library, we have performed a two-way ANOVA analysis. We set a threshold of p < 0.05 for statistical significance. The proliferation assays were performed in eight technical replicates and the migration assays in four technical replicates.

### 2.7 Data preparation for regression tasks

Both proliferation data and migration data were processed prior to regression modelling and to ensure comparability between experiments.

Specifically, the proliferation data was normalized to the mean value of all 8 replicates at a Mg concentration of 0.8 mM for any donor and passage number, respectively. Therefore, where individual measurements exceeded those of the mean, proliferations above 100 % were recorded. Overall, 11 datasets were available, as passages 4,5,7 had been assessed for donor 1, donor 2 and the donor pool, yet data for passage 6 was available for donor 1 and donor 2 only. Following normalization, feature engineering was performed for five different regression tasks: (i) prediction of one missing concentration within a passage, (ii) prediction of two missing concentrations within a passage, (iii) prediction of three missing concentrations within a passage, (iv) prediction of a missing passage for a given donor, and (v) prediction of a missing donor for a given passage. In all cases, the data for any given donor and passage at a certain Mg concentration was shuffled randomly to avoid the regression models learning a specific error based on an assumed dependence on the well plate row. In (i)-(iii), the split of the datasets into training, validation and test was 8/2/1. Further, the data was split into features and targets such that the missing concentrations were predicted based on the remaining predictions. For example, in case (i) the target value was the proliferation at that missing concentration. The input values for this prediction were the proliferation at all other concentrations. All cases of possible missing concentrations were assessed As there were multiple possibilities in the cases (ii) and (iii) where two or three concentrations were missing, respectively, all potential combinations were used to assess the mean leave-out-error for the different regression models. The passage number and donor number (categorical) were also used as features. In the case of (iv), where a missing passage for a given donor was predicted based on the other two passages, only the data from passages 4, 5 and 7 were used. The target was the proliferation at the missing passage, while the features were given as the proliferation at the other passages, the donor number (categorical) and the Mg concentration. In this case, the split into training/validation/test was such that the test dataset was all data from a given donor, while three concentrations of one passage of one of the other donors were retained for validation from the remaining data. For the prediction of the proliferation of cells for a missing donor (v) and a given passage, the data was again limited to passages 4, 5 and 7. In this case, the target was the proliferation for a missing donor, and the features were defined as the proliferation for the other donors, the passage number and the Mg concentration. The training/validation/test split was performed as in case (iv). In all cases, the data for every configuration was saved, to ensure that all regression models were validated and tested with the same dataset. This was required due to the column shuffling.

### 2.8 Baselines

Two simple baseline methods were tested to provide reference points for model performance. The first, which we call label averaging, predicts missing labels by computing the mean of the corresponding labels across the training set. The second, which we call data averaging, estimates missing values within a sample by averaging the values of adjacent concentrations or passages. These baselines require no model training and serve to contextualize the predictive power of more complex approaches.

### 2.9 Linear regression, support vector regression, random forest regression

Four simple regression models were implemented using scikit-learn in Jupyter Notebooks with Python 3 to test their suitability to predict HUVEC proliferation and migration. Specifically, linear regression (LR), support vector regression (SVR) with linear and radial basis function (RBF) kernel, and random forest (RFR) regression were used. During the validation step, a grid search was performed to optimize the respective hyperparameters. These were the penalty parameter C and epsilon for SVR and number of trees and their maximum depth for RF. The optimal hyperparameters are given in Table S1 in the supplementary information. The best hyperparameter set was defined by the lowest mean absolute error (MAE) for the validation sets. All computations were performed on a standard issue 64-bit Windows laptop.

### 2.10 Neural networks

The described regression tasks were also modelled using feed-forward neural networks, implemented in PyTorch with Python 3.9. A comprehensive Bayesian hyperparameter search was conducted for each task. The optimal hyperparameter settings identified were very similar across all tasks. Therefore, the same values, as listed in Table S2 in the appendix, were applied to all tasks. For each task, prediction errors were averaged over all tested formulations, thereby enhancing the robustness of the results.

### 2.11 Large language models

Large Language Models (LLMs) are typically employed for text-related tasks such as text generation or summarization. Testing a state-of-the-art LLM's ability to solve these specific regression tasks appeared intriguing. Training data can be provided to an LLM in two ways: as examples (few-shots) within the prompt or by fine-tuning the model on the data. Given the small number of data points, providing them as examples within the prompts seemed sufficient. Additionally, this approach allowed the use of OpenAI's most recently released reasoning model, o4-mini, for which fine-tuning is not yet permitted [19]. Furthermore, we tested the established GPT-4o as LLM baseline. The process of obtaining predictions for missing or test data is illustrated in Figure S1 in the appendix. The prompts that include the training data and request the LLMs’ predictions on the test data are detailed in Table S3 in the appendix. It proved beneficial to split the requests for predictions on the test data into smaller batches of eight data points each. Each batch of test data involves a new call to the model. This batching structure was also applied to the presentation of the training data: chunks of eight training data points were provided as examples sequentially. With a few exceptions, the model successfully delivered the predictions in valid JSON format. The few errors in the prediction format were corrected manually.

### 2.12 Statistics

To assess the statistical significance in the performance of the different models, which have been implemented, we have implemented a Welch’s *t*-test in Python for a pair-wise comparison of the models.

## 3 Results and discussion

Angiogenesis is a phenomenon that includes different steps, such as endothelial cell proliferation, differentiation, and migration. Finally new blood vessels will be developed in order to ensure tissue regeneration by supply of nutrients and oxygen as well as regenerative cell recruitment. Although an angiogenetic effect of Mg was proven the mechanisms behind this induction have to be elucidated [7]. The HUVECs provide a suitable model system to study angiogenic components on a functional level. Endothelial cells form a thin layer within blood vessels and play a pivotal role as barrier between blood stream and surrounding soft tissue. But this cell type allows also the diffusion of metabolites and the passaging of immune cells. Insofar endothelial cells provide distinct characteristics and HUVECS are a very good source for a cell model expressing the targeted characteristics of proliferation and migration [20].

### 3.1 HUVEC proliferation

**Fig. 1:**
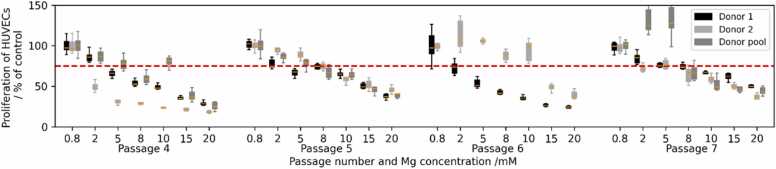
Proliferation of HUVEC in % of control for passages 4, 5, 6 and 7 and donor 1 (black), donor 2 (light grey) and donor pool (dark grey) at different Mg concentrations. The red dashed line indicates 75 %.

Fig. 1 displays the proliferation of HUVECs for donor 1, donor 2 and the donor pool as well as passages 4–7. While the overall response is similar, i.e. the proliferation decreases with increasing concentration of Mg, there are some deviations between the responses, in particular for donor 2 in passages 4 and 6 and the donor pool in passage 7. Based on the results, it appears that depending on the passage number, a concentration between 2 and 5 mM results in a proliferation of 75 % or below, which we consider the threshold for viability.

The two-way ANOVA test reveals that passage number and Mg concentration have a significant effect on the HUVEC proliferation (p < 0.05), while the donor type does not.

Although a concentration dependent effect on cell proliferation was expected, the sensitivity of all used HUVEC sources was high. Concentrations above 2 mM Mg already induce a loss of proliferation capacity and 10 mM Mg, a concentration which is recommended by other references, was not tolerated. It is known that Mg might influence proliferation assays that are based on the mitochondrial dehydrogenase to produce coloured formazan derivatives [21]. Here, a CCK8 test was applied also dependent on enzyme activities but we normalized the measured values to the related Mg containing cell culture medium without cells. In general, the application of different assays types increases the challenge of comparability.

The gene expression analysis was performed with proliferating cells to demonstrate adaptations in the cellular processes in dependency of Mg exposure. The RNA yield of lysed cells are reduced with increasing Mg concentrations with both donors. The yield summarized over all tested cell passages decreased from 707 ng/mL (+/- 120 ng/mL) in the control to 497 ng/mL (+/- 86 ng/mL) in the 5 mM Mg treatment group to 330 ng/mL (+/- 141 ng/mL) in the 10 mM Mg treatment group, respectively for donor 1 and similarly for donor 2 from 680 ng/mL (+/-120 ng/mL) in the control to 290 ng/mL (+/- 52 ng/mL) and 172 ng/mL (+/- 27 ng/mL) for 5 and 10 mM Mg. These data reflect the susceptibility of the primary HUVEC preparations against Mg of 5 mM and 10 mM in this approach. According to the previous work by Xu et al. a concentration-dependent upregulation of the Vascular endothelial growth factor receptor 2 (KDR) was found. The expression of this gene was significantly upregulated by 2-fold. A comparable upregulation was also observed for Vascular endothelial growth factor receptor 1 (FLT1) but only for donor 2. The expression of the two angiogenic factors VEGF 1 and VEGF 2 was not significantly influenced, in contrast to the observations by Xu et al. The expression ANG was not changed by donor 1 but reduced by donor 2. The data indicate that Mg induces the expression of growth factor receptors at the surface of endothelial cells. However, the type and amount of data, the donor dependency and the low sensitivity of expression change did not allow the utilization of this approach for simulation and modelling.

### 3.2 Migration

**Fig. 2:**
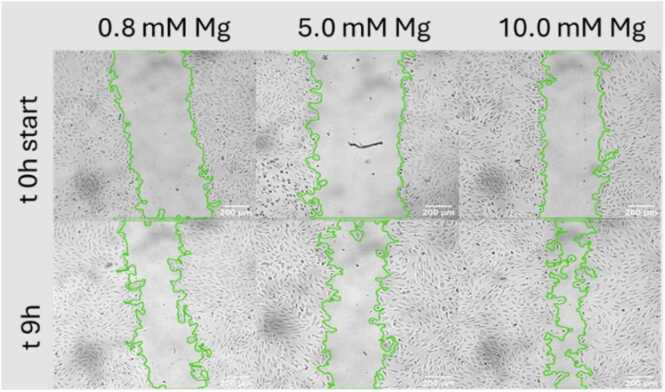
Example of microscopic images of the scratch test for HUVECs (donor 1, passage 7) subjected to different Mg concentrations. The area outlined in green is the cell-free area based on which the wound closure is computed.

The migration assays were performed to demonstrate the ability of endothelial cells to migrate and consequently elongate newly formed vessels including sprouting. Therefore, the proliferation was suppressed by starvation and mitomycin. This was necessary to suppress the influence of cell proliferation in the wound healing and to identify pure cell migration [22]. Mitomycin is a DNA synthesis inhibitor successfully applied for cancer therapy [23]. The microscopic images (Fig. 2) showed the closure of the scratch/wound area over time and furthermore cells developing filopodia have been found. The data evaluation considered the percentage of areas without cells. Although the cells were sensitive to 5 and 10 mM Mg supplementation the short term exposure for 9 h during the imaging was tolerated.

**Fig. 3:**
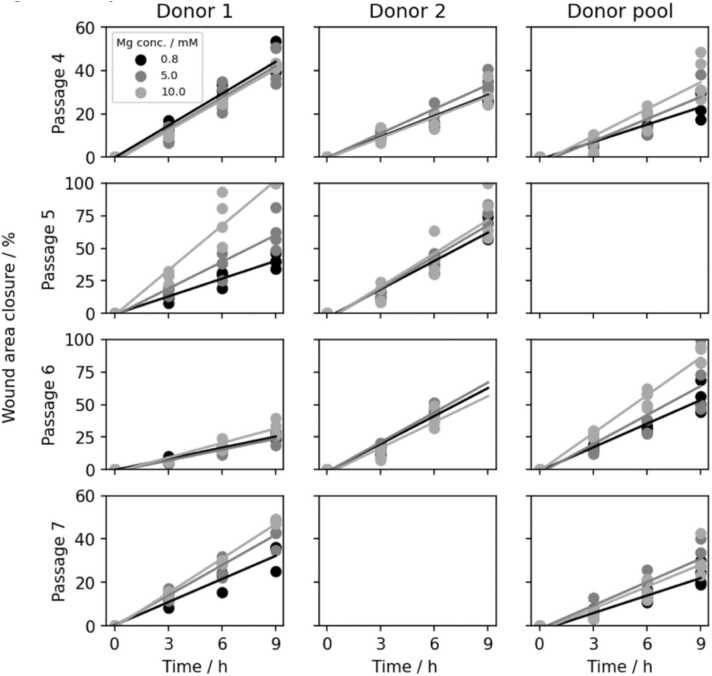
Wound area closure in % over 9 h to display HUVEC migration for passages 4, 5, 6 and 7 and donor 1, donor 2 and donor pool at different Mg concentrations.

Fig. 3 shows wound area closure computed from microscopic images and fitted linear slopes for all passages and donors. Empty plots relate to missing experiments. It is apparent that the cell migratory behaviour deviates between donors and passage numbers. Higher migration capacities are found at passage 5 and passage 6 and the addition of Mg generally increases this capacity. The effect of Mg addition appears lowest for donor 2 for all passage numbers, which coincides with the inconsistencies in behaviour of this donor for cell proliferation as well. Donor 1 shows a non-linear affect with respect to passage numbers, similar also to the effects observed for proliferation. The ambiguous effect of Mg addition on HUVEC migration capacity agrees with the literature, where concentrations of 2 mM only were shown to have an increasing effect [6]. Higher concentrations were not found to differ significantly from the control.

### 3.3 Regression results

**Fig. 4:**
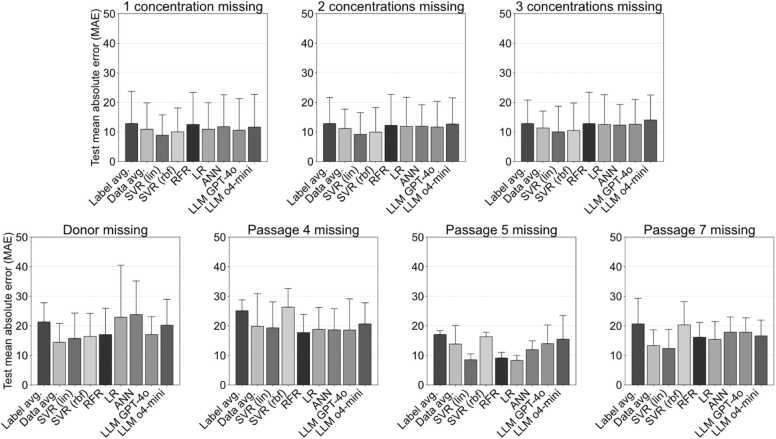
Mean absolute error (MAE) for test data for cases (I)-(v), with case (iv) divided per predicted passage. The results are given as mean ± standard deviation for all regression models tested: support vector regression (SVR – with linear or rbf kernel), random forest regression (RFR), linear regression (LR), neural networks (ANN) and the large language models GPT-4o and o4-mini. additionally, we introduce two simple baseline methods: label averaging, which predicts each missing label as the average of the corresponding labels in the training data; and data averaging, which uses only the available data from the current sample to predict missing values by averaging the preceding and succeeding concentrations or passages.

Fig. 4 shows the computed test MAEs for the regression of HUVEC proliferation for cases (i)-(v) and the regression models tested. The statistical significance in displayed in supplementary tables 4–10. While the standard deviations are high, there is a trend for lower mean testing MAEs for traditional regression models over LLMs. Specifically, the linear kernel SVR appears to perform best in the majority of cases. A mean error around 10 % can be achieved when information on the proliferation of HUVEC for up to three concentrations is missing, which may be deemed acceptable given the large deviation in experimental data. Similarly, the proliferation of whole passages can be predicted with errors as low as 8.5 ± 2.0 % for passage 5. While the performance differences between methods remain small relative to the overall prediction variance, the linear kernel SVR, identified as the best-performing regression model, consistently outperforms the simple baselines. These differences are significant for the label avg. baseline in all cases, as well as the data avg. for case (iv) if passage 5 was missing and cases (ii) and (iii). In contrast, the large and complex LLMs perform approximately on par with these baselines. This suggests that a certain level of predictive accuracy can be achieved through very simple approaches, and that further improvements through extensive model architecture design and hyperparameter tuning yield only small gains.

**Fig. 5:**
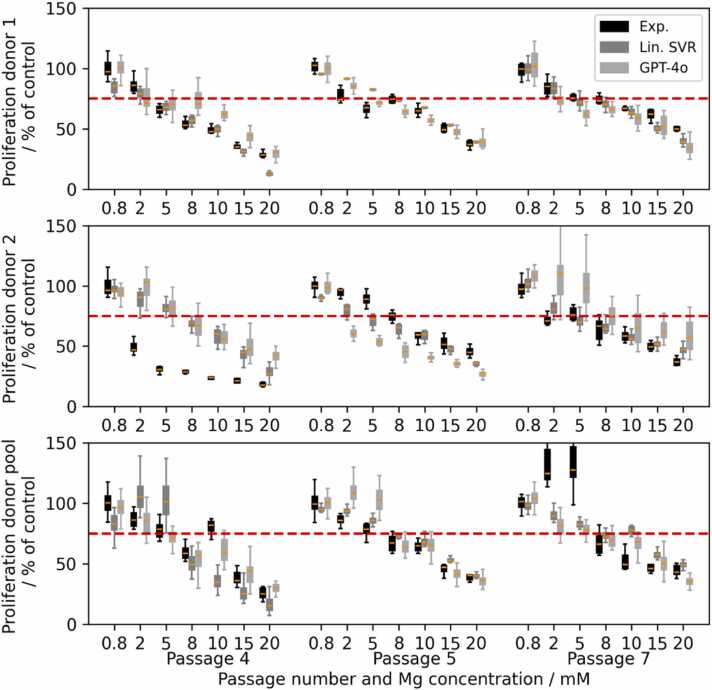
Experimental data (black) and prediction from the best linear kernel SVR (dark gray) and LLM GPT-4o (light gray) for the passages tested in case (iv) of the regression of HUVEC proliferation. The red dashed line indicates 75 %.

To consider the differences between the predicted test passages for linear SVR and LLM in detail, Fig. 5 displays the results of these for case (iv) in comparison to the experimental data for each donor and passages 4, 5 and 7. As seen in the figure, the linear SVR results in predictions that show a steady decline in proliferation with increasing Mg concentration with little deviation except for donor 1 passage 7 and donor pool passage 4. By contrast, the LLM prediction appears more susceptible to deviations in the training data. Moreover, considering the difference between experimental results and regression shown for donor 2 passage 4 in Fig. 5, it becomes apparent that the high MAEs and standard deviations shown in Fig. 4 are partially due to inconsistencies in the cell behaviour depending on different donors. This is also the reason, why the mean error for predicting the proliferation of cells of a certain donor at a given passage is higher at 15.7 ± 8.6 %.

Therefore, as it is well known, our study highlights that the amount of consistent data is crucial in attaining low testing errors. Overall, the number of data used in this regression study can be considered low with 77 distinct datasets of varying concentration, passage number, and donor and eight repeats for each. Nevertheless, the relative error achieved in this study is comparable to that reported in the literature. Mehrian et al. [14] trained their regression model on the information from 131 donors, after removing missing information and outliers, and for four different passage numbers. They achieved a test MAE between 0.7 and 0.87, while their target values ranged between 0 and 7 mostly, equating to a relative test MAE in a similar range as the one achieved in our study. The same holds true for the study of Shen et al. [15], who trained on 272 data sets and reported a test MAE of 0.22 for target values between 0 and 3. To achieve better prediction results, one might simplify the prediction task to a classification rather than a regression problem. Rafieyan et al. reported accuracies of 87 % in classifying the cell response with respect to different scaffold material compositions [24]. In a similar manner, the current regression task could be simplified to a positive/negative classification problem, by considering all proliferation below 75 % as negative.

## 4 Conclusion and outlook

The data-driven assessment of the performance of regression models and LLM highlight the importance of utilizing multiple cell culture tests with cells from different donors and at different passage numbers. Magnesium as degradable metallic implant was used as a demonstration case because of its therapeutic potential. The generally available research data provide only a patchy synopsis because of a lack of methodological homogeneity and data gaps. As such, regression models can be trained to fill in gaps in the data and obtain predictions that are less biased by irregular behaviour for certain donors. The most state-of-the-art LLMs perform well in comparison to traditional regression models, with the latter being less influenced by irregularities. Future work should focus on including more data from the literature for benchmarking and further testing the capabilities of the LLMs. It should also consider the potential benefit of combining several different predictors into an ensemble in order to finetune the results.

## CRediT authorship contribution statement

**Sven Tomforde:** Writing – review & editing, Funding acquisition. **Roland Aydin:** Writing – review & editing, Supervision, Conceptualization. **Regine Willumeit-Römer:** Writing – review & editing, Conceptualization. **Berit Zeller-Plumhoff:** Writing – original draft, Supervision, Software, Investigation, Funding acquisition, Conceptualization. **Heike Helmholz:** Writing – original draft, Supervision, Funding acquisition, Formal analysis, Conceptualization. **Redon Resuli:** Investigation, Formal analysis. **Marius Tacke:** Writing – original draft, Software, Investigation. **Jalil Nourisa:** Software, Funding acquisition.

## Declaration of Competing Interest

The authors declare that they have no known competing financial interests or personal relationships that could have appeared to influence the work reported in this paper.

## Data Availability

All data is available from the authors upon reasonable request.
