# Supplementary material for: Regression models for the prediction of the influence of magnesium ions on primary endothelial cell (HUVEC) proliferation and migration
Source: Comput Struct Biotechnol J. 2025 Jun 8;27:2711–8. doi: 10.1016/j.csbj.2025.06.023 (PMC12241815; doi:10.1016/j.csbj.2025.06.023)
Supplement: Supplementary file 1 — Supplementary material [file mmc1.docx]

**Appendix**

| Regression model | Hyperparameter | Optimal setting for case | | | | | | |
| --- | --- | --- | --- | --- | --- | --- | --- | --- |
|  |  | (i) | (ii) | (iii) | (iv) – p4 | (iv) – p5 | (iv) – p7 | (v) |
| Support vector regression with linear kernel | Regularization parameter $C$ | 0.1 | 0.01 | 0.01 | 15 | 1 | 0.01 | 0.01 |
|  | $\varepsilon$ - tube | 0.01 | 0.001 | 0.001 | 20 | 0.1 | 0.01 | 0.01 |
| Support vector regression with rbf kernel | Regularization parameter $C$ | 50 | 50 | 50 | 5000 | 500 | 5 | 50 |
|  | $\varepsilon$ - tube | 0.5 | 0.05 | 0.05 | 5 | 0.5 | 0.1 | 0.05 |
| Random forest regression | Numer of estimators | 100 | 200 | 100 | 10 | 10 | 20 | 20 |
|  | Max. tree depth | 2 | 2 | 2 | 20 | 200 | 5 | 10 |

Table S1: Optimized hyperparameters for support vector regression and random forest regression.

| Hyperparameter | Setting |
| --- | --- |
| Activation function | Leaky ReLU |
| Layers | 3 |
| Neurons per Layer | 8 |
| Batch size | 4 |
| Optimizer | Stochastic Gradient Descent (SGD) |
| Learning rate scheduler | ReduceLROnPlateau (PyTorch) |
| Learning rate | 0.04 |
| Learning rate decay factor | 0.5 |
| Learning rate decay patience | 20 |
| Momentum | 0.6 |
| Early stopping patience | 20 |
| Min epochs to train | 50 |
| Max epochs to train | 500 |

Table S2: Hyperparameter settings of Feedforward Neural Network


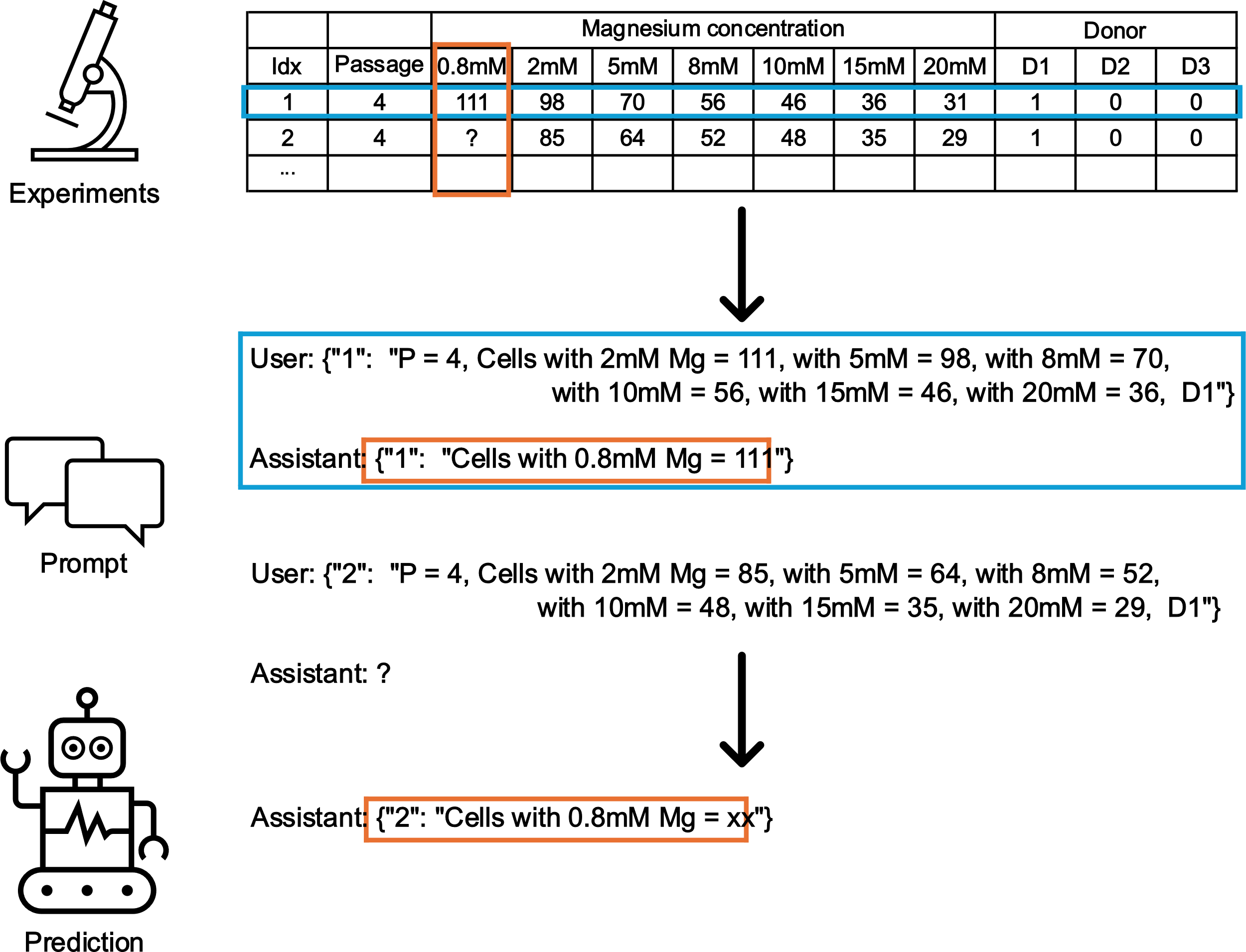


Figure S1: Flowchart illustrating the process of obtaining predictions for missing or test data points using a Large Language Model.

| Type | | Content |
| --- | --- | --- |
| Context | | “Act as a biologist. Your task is to predict the proliferation of human umbilical vein endothelial cells (HUVECs) dependent on their passage and the concentration of a magnesium infusion to the culturing medium. Each sample of the corresponding experiments consists of the cell passage (P), the relative number of cells counted after culturing with a specified magnesium concentration (number of cells with xx mM Mg) and whether the cells come from Donor 1 (D1), Donor 2 (D2) or Donor 3 (D3). Here are some examples:” |
| Message | Training data (Examples) | “0: P = 4, number of cells with 2 mM Mg = 52.625,  with 5 mM Mg = 32.771, with 8 mM Mg = 33.661,  with 10 mM Mg = 22.605, with 15 mM Mg = 22.784,  with 20 mM Mg = 16.789, D2”  …  ”0: Number of cells with 0.8 mM Mg = 115.377” |
|  | Test data | “These were the examples. For the following samples, predict the relative number of cells with a magnesium concentration of 0.8 mM.”  ”72: P = 5, number of cells with 2 mM Mg = 78.356,  with 5 mM Mg = 97.381, with 8 mM Mg = 68.895,  with 10 mM Mg = 57.003, with 15 mM Mg = 43.666,  with 20 mM Mg = 51.946, D2” |
|  | Final text | “Predict the proliferation of human umbilical vein endothelial cells (HUVECs) dependent on their passage and the concentration of a magnesium infusion to the culturing medium. Provide your predictions in the following json format: ‘72’: ‘Number of cells with 0.8 mM Mg = xx’, ‘73’: ‘Number of cells with 0.8 mM Mg = xx’, ‘74’: ‘Number of cells with 0.8 mM Mg = xx’, .... Base your predictions on the provided examples and on your theoretical knowledge. Take your time and think about your predictions step-by-step. If you are unsure, just let me know your best guess. Your contribution is highly appreciated.” |

Table S3: Exemplary prompt used with OpenAI’s GPT-4o

| **Model** | **Label avg.** | **Data avg.** | **SVR (lin)** | **SVR (rbf)** | **RFR** | **LR** | **ANN** | **GPT-4o** | **o4-mini** |
| --- | --- | --- | --- | --- | --- | --- | --- | --- | --- |
| **Label avg.** | - |  | * |  |  |  |  |  |  |
| **Data avg.** |  | - |  |  |  |  |  |  |  |
| **SVR (lin)** |  |  | - |  | * |  | * |  |  |
| **SVR (rbf)** |  |  |  | - |  |  |  |  |  |
| **RFR** |  |  |  |  | - |  |  |  |  |
| **LR** |  |  |  |  |  | - |  |  |  |
| **NN** |  |  |  |  |  |  | - |  |  |
| **GPT-4o** |  |  |  |  |  |  |  | - |  |
| **o4-mini** |  |  |  |  |  |  |  |  | - |

Table S4: Statistical significance of Welch’s t-test for 1 concentration missing. * indicates p<0.05.

| **Model** | **Label avg.** | **Data avg.** | **SVR (lin)** | **SVR (rbf)** | **RFR** | **LR** | **NN** | **GPT-4o** | **o4-mini** |
| --- | --- | --- | --- | --- | --- | --- | --- | --- | --- |
| **Label avg.** | - | * | * | * |  |  |  |  |  |
| **Data avg.** |  | - | * |  |  |  |  |  | * |
| **SVR (lin)** |  |  | - |  | * | * | * | * | * |
| **SVR (rbf)** |  |  |  | - | * | * | * | * | * |
| **RFR** |  |  |  |  | - |  |  |  |  |
| **LR** |  |  |  |  |  | - |  |  |  |
| **ANN** |  |  |  |  |  |  | - |  |  |
| **GPT-4o** |  |  |  |  |  |  |  | - |  |
| **o4-mini** |  |  |  |  |  |  |  |  | - |

Table S5: Statistical significance of Welch’s t-test for 2 concentration missings. * indicates p<0.05.

| **Model** | **Label avg.** | **Data avg.** | **SVR (lin)** | **SVR (rbf)** | **RFR** | **LR** | **ANN** | **GPT-4o** | **o4-mini** |
| --- | --- | --- | --- | --- | --- | --- | --- | --- | --- |
| **Label avg.** | - | * | * | * |  |  |  |  | * |
| **Data avg.** |  | - | * |  | * | * | * | * | * |
| **SVR (lin)** |  |  | - |  | * | * | * | * | * |
| **SVR (rbf)** |  |  |  | - | * | * | * | * | * |
| **RFR** |  |  |  |  | - |  |  |  |  |
| **LR** |  |  |  |  |  | - |  |  | * |
| **ANN** |  |  |  |  |  |  | - |  | * |
| **GPT-4o** |  |  |  |  |  |  |  | - | * |
| **o4-mini** |  |  |  |  |  |  |  |  | - |

Table S6: Statistical significance of Welch’s t-test for 3 concentrations missing. * indicates p<0.05.

| **Model** | **Label avg.** | **Data avg.** | **SVR (lin)** | **SVR (rbf)** | **RFR** | **LR** | **ANN** | **GPT-4o** | **o4-mini** |
| --- | --- | --- | --- | --- | --- | --- | --- | --- | --- |
| **Label avg.** | - |  |  |  | * | * | * |  |  |
| **Data avg.** |  | - |  |  |  |  |  |  |  |
| **SVR (lin)** |  |  | - | * |  |  |  |  |  |
| **SVR (rbf)** |  |  |  | - | * | * | * | * |  |
| **RFR** |  |  |  |  | - |  |  |  |  |
| **LR** |  |  |  |  |  | - |  |  |  |
| **ANN** |  |  |  |  |  |  | - |  |  |
| **GPT-4o** |  |  |  |  |  |  |  | - |  |
| **o4-mini** |  |  |  |  |  |  |  |  | - |

Table S7: Statistical significance of Welch’s t-test for Passage 4 missing. * indicates p<0.05.

| **Model** | **Label avg.** | **Data avg.** | **SVR (lin)** | **SVR (rbf)** | **RFR** | **LR** | **ANN** | **GPT-4o** | **o4-mini** |
| --- | --- | --- | --- | --- | --- | --- | --- | --- | --- |
| **Label avg.** | - |  | * |  | * | * | * |  |  |
| **Data avg.** |  | - | * |  | * | * |  |  |  |
| **SVR (lin)** |  |  | - | * |  |  | * | * | * |
| **SVR (rbf)** |  |  |  | - | * | * | * |  |  |
| **RFR** |  |  |  |  | - |  | * | * | * |
| **LR** |  |  |  |  |  | - | * | * | * |
| **ANN** |  |  |  |  |  |  | - |  |  |
| **GPT-4o** |  |  |  |  |  |  |  | - |  |
| **o4-mini** |  |  |  |  |  |  |  |  | - |

Table S8: Statistical significance of Welch’s t-test for Passage 5 missing. * indicates p<0.05.

| **Model** | **Label avg.** | **Data avg.** | **SVR (lin)** | **SVR (rbf)** | **RFR** | **LR** | **ANN** | **GPT-4o** | **o4-mini** |
| --- | --- | --- | --- | --- | --- | --- | --- | --- | --- |
| **Label avg.** | - | * | * |  |  |  |  |  |  |
| **Data avg.** | * | - |  | * |  |  | * | * |  |
| **SVR (lin)** |  |  | - | * |  |  | * | * |  |
| **SVR (rbf)** |  |  |  | - |  |  |  |  |  |
| **RFR** |  |  |  |  | - |  |  |  |  |
| **LR** |  |  |  |  |  | - |  |  |  |
| **ANN** |  |  |  |  |  |  | - |  |  |
| **GPT-4o** |  |  |  |  |  |  |  | - |  |
| **o4-mini** |  |  |  |  |  |  |  |  | - |

Table S9: Statistical significance of Welch’s t-test for Passage 7 missing. * indicates p<0.05.

| **Model** | **Label avg.** | **Data avg.** | **SVR (lin)** | **SVR (rbf)** | **RFR** | **LR** | **ANN** | **GPT-4o** | **o4-mini** |
| --- | --- | --- | --- | --- | --- | --- | --- | --- | --- |
| **Label avg.** | - | * | * | * | * |  |  | * |  |
| **Data avg.** |  | - |  |  |  |  | * | * | * |
| **SVR (lin)** |  |  | - |  |  | * | * |  | * |
| **SVR (rbf)** |  |  |  | - |  | * | * |  |  |
| **RFR** |  |  |  |  | - |  | * |  |  |
| **LR** |  |  |  |  |  | - |  |  |  |
| **ANN** |  |  |  |  |  |  | - | * |  |
| **GPT-4o** |  |  |  |  |  |  |  | - |  |
| **o4-mini** |  |  |  |  |  |  |  |  | - |

Table S10: Statistical significance of Welch’s t-test for one donor missing. * indicates p<0.05.
